# Pathogenic bacteria significantly increased under oxygen depletion in coastal waters: A continuous observation in the central Bohai Sea

**DOI:** 10.3389/fmicb.2022.1035904

**Published:** 2022-11-21

**Authors:** Yiyan Guo, Chao Wu, Jun Sun

**Affiliations:** ^1^Research Centre for Indian Ocean Ecosystem, Tianjin University of Science and Technology, Tianjin, China; ^2^Key Laboratory of Sustainable Development of Marine Fisheries, Ministry of Agriculture and Rural Affairs, Yellow Sea Fisheries Research Institute, Chinese Academy of Fishery Sciences, Qingdao, China; ^3^Laboratory for Marine Fisheries Science and Food Production Processes, Pilot National Laboratory for Marine Science and Technology, Qingdao, China; ^4^Institute for Advanced Marine Research, China University of Geosciences, Guangzhou, China; ^5^State Key Laboratory of Biogeology and Environmental Geology, China University of Geosciences (Wuhan), Wuhan, China

**Keywords:** pathogenic bacteria, hypoxia, the Bohai Sea, high-throughput sequencing, bacterial communities

## Abstract

The spread of pathogenic bacteria in coastal waters endangers the health of the local people and jeopardizes the safety of the marine environment. However, their dynamics during seasonal hypoxia in the Bohai Sea (BHS) have not been studied. Here, pathogenic bacteria were detected from the 16S rRNA gene sequencing database and were used to explore their dynamics and driving factors with the progressively deoxygenating in the BHS. Our results showed that pathogenic bacteria were detected in all samples, accounting for 0.13 to 24.65% of the total number of prokaryotic sequences in each sample. Pathogenic *Proteobacteria* was dominated in all samples, followed by *Firmicutes*, *Actinobacteria*, *Tenericutes*, and *Bacteroidetes*, etc. β-diversity analysis showed that pathogenic bacteria are highly temporally heterogeneous and regulated by environmental factors. According to RDA analysis, these variations may be influenced by salinity, ammonia, DO, phosphate, silicate, and Chl a. Additionally, pathogenic bacteria in surface water and hypoxia zone were found to be significantly separated in August. The vertical distribution of pathogenic bacterial communities is influenced by several variables, including DO and nutrition. It is noteworthy that the hypoxia zones increase the abundance of certain pathogenic genera, especially *Vibrio* and *Arcobacter*, and the stability of the pathogenic bacterial community increased from May to August. These phenomena indicate that the central Bohai Sea is threatened by an increasingly serious pathogenic community from May to August. And the developing hypoxia zone in the future may intensify this phenomenon and pose a more serious threat to human health. This study provides new insight into the changes of pathogenic bacteria in aquatic ecosystems and may help to make effective policies to control the spread of pathogenic bacteria.

## Introduction

Pathogenic bacteria generally refer to those microorganisms that can cause infection or even infectious diseases in human. Nowadays, pathogenic bacteria attract extensive attention worldwide due to their higher pathogenicity and/or lethality (Pond, 2013). Pathogenic bacteria not only exist in the body of humans and animals, but also widely distributed in various environmental mediums such as livestock, aquaculture, wastewater, natural waters, and so on (Ryu et al., 2012; Done et al., 2015; Sun et al., 2016; Tang et al., 2016). Recently, increasing studies demonstrated that marine environment is a key hotspot to harbor pathogenic bacteria (Leight et al., 2018; Ferchichi et al., 2021; Thorstenson and Ullrich, 2021). The transmission of pathogenic bacteria occurs frequently in the water environment (Gomi et al., 2015). Humans can become unwell when they are exposed to or drink contaminated water (Botzenhart and Seidel, 2010). Meanwhile, people are vulnerable to infection with pathogenic bacteria due to improper raw seafood or cooking. Seafood products grown in polluted waters, especially filter-feeding bivalves/mollusks such as oysters, mussels, clams, and arks, can concentrate bacteria or viruses from the polluted waters (Shuval, 2003). According to literature, the most common pathogenic bacteria in marine environment including *Escherichia coli O157*, *Salmonella*, *Campylobacter*, *Vibrio traumatica*, et al. (Pond, 2013).

Most pathogenic bacteria are not native microorganisms but come from exogenous contamination (Pandey et al., 2014). Land-based pollutants can bring pathogenic bacteria into the seawater, causing a variety of infectious diseases (Shuval, 2003). More seriously, the abuse and excessive discharge of antibiotics have led to an increase in the drug resistance of pathogenic bacteria, such as “NDM-1” with resistance to most antibiotics (Davies, 1994; Walsh et al., 2011). Pathogenic bacteria are important components of bacterial communities in the natural environment. Bacteria and the environment are interdependent and mutually constrained under certain conditions (Liu et al., 2012). Temperature, pH, salinity, dissolved oxygen (DO), and other environmental conditions all can impact the distribution and structure of microorganisms in marine (Yu et al., 2018). DO is one of the most critical environmental elements controlling the distribution and organization of marine bacterial communities (Zhao et al., 2021). The diversity, abundance, and function of microbial communities have been found to be affected by DO concentrations (Ye et al., 2016; Jessen et al., 2017). Interestingly, the abundance of *Vibrio* in water has been demonstrated to be adversely associated with DO or pH in several investigations (Kaper et al., 1979; Rehnstam-Holm et al., 2013). Other physical and chemical variables also have improved to influence the growth and dispersion of these bacteria as well (Kaper et al., 1979). In contrast, no study has investigated the direct influence of hypoxia and acidification on pathogenic bacteria communities in the coastal environment.

In recent years, the emergence and expansion of oceanic hypoxia zones have been continuously reported in coastal oceans (Breitburg, 2002). Hypoxia is commonly characterized as a DO content in water that is less than 3.01 mg O_2_/L (Rabalais et al., 2010). The hypoxia zone also was reported existing in the Bohai Sea, which is a semi-enclosed sea of China with an average depth of 18 m. The Bohai Sea is characterized by infrequent exchange with the Yellow Sea and a water residence time of ~1.5 years (Li et al., 2015). It was widely believed that the effects of seasonal stratification and organic matter remineralization together caused the summers hypoxia in the BHS (Zhao et al., 2017; Zhai et al., 2019). For most coastal oceans, eutrophication first leads to increased organic matter in the sediment, subsequently promoting microbial growth and respiration (Wei et al., 2019). Remineralization through oxygen-consuming processes ultimately leads to greater oxygen demand. Hypoxia will occur if bottom DO cannot be restored by local hydrodynamics (Diaz and Rosenberg, 2008). Meanwhile, hypoxia is usually accompanied by the occurrence of localized acidification. Large amounts of CO_2_ were produced during the mineralization and decomposition of organic matter, which lowers the pH of hypoxia zone (Zhai et al., 2012). As a semi-enclosed sea, the Bohai Sea tends to form regular seasonal hypoxia zone and marine acidification. Accordingly, this coastal region is an ideal model system to study the distribution of pathogenic bacteria along spatial and physicochemical gradients and unveil relative influences of hypoxia and acidification.

Here, we conducted the first detailed survey of pathogenic bacterial communities in the seasonal hypoxia zone of the Bohai Sea from May to August 2017. The effects of hypoxia and acidification on the spatial and temporal distribution of pathogenic bacterial communities were investigated using a high-throughput sequencing approach based on 16S rRNA genes. Our specific goals were to (1) evaluate the community structure and diversity of pathogenic bacteria; (2) explore whether changes in DO and pH are accompanied by a shift in the community composition of pathogenic bacteria; (3) uncover the interactions between pathogenic bacteria and other bacteria by constructing microbial co-occurrence networks; (4) further evaluate the role of other environmental factors in controlling the shape of pathogenic bacterial community structure in seasonal anoxic acidification zones. This study provides a comprehensive understanding of the changes in the community structure of pathogenic bacteria and provides guidance for further research on coastal hypoxia and biological effects in the Bohai Sea.

## Materials and methods

### Study location, sample collection, and analysis

**Figure 1:**
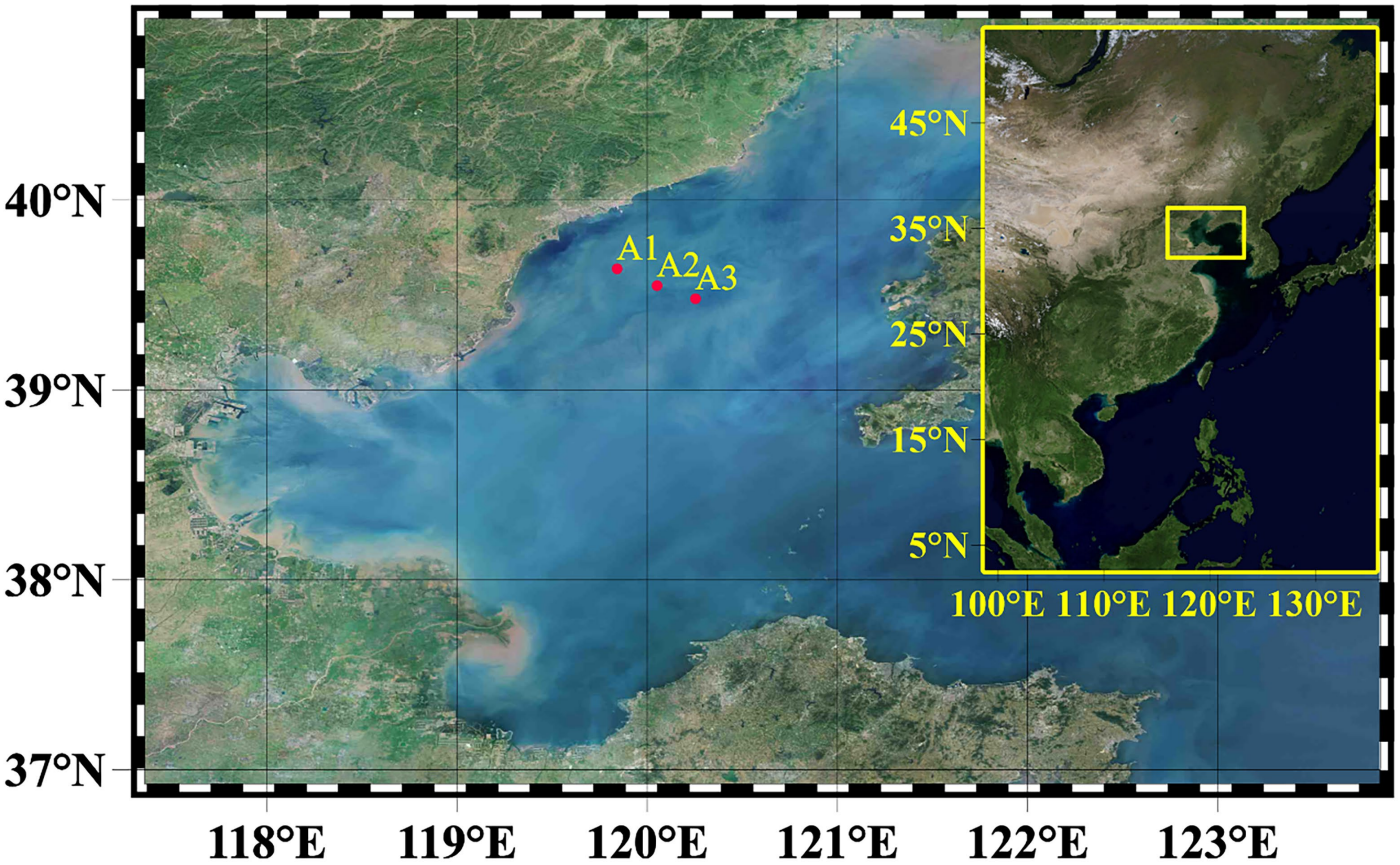
The map showing the sampling stations in the Bohai Sea (BHS). In summary, samples from 3 sites (A1–A3) were used for molecular analysis.

From May to August 2017, water samples were taken during four consecutive monthly cruises from three stations (A1–A3) in the Bohai Sea’s shelf region (Figure 1). Vertical samples were collected at all stations using a Niskin water sampler with an RBR620 multi-sensor to assess environmental parameters from 2 to 5 depths. In August, a significant deoxygenation of the bottom at points A1 to A3 was observed. Finally, 46 DNA samples were collected for high-throughput sequencing at these three stations from May to August 2017. The detailed sample sources are May (A2: 1, 15, 25 m, A3: 1, 15, 25 m), June (A1: 1, 6, 12, 18 m, A2: 1, 10, 18 m, A3: 1, 7, 13, 18, 21 m), July (A1. 1, 5, 15, 18 m, A2: 1, 5, 10, 18, 22 m, A3: 1, 6, 12, 19, 22 m), August (A1: 1, 6, 12, 18 m, A2: 1, 5, 12, 18, 22 m, A3: 1, 6, 12, 19, 22 m). Due to traffic control, station A1 samples were missing in May.

DO and pH sensors were calibrated according to standard protocols prior to each cruise (Luo et al., 2016). Collected seawater samples were transferred to separate 10 l PE basins (pre- rinsed with 10% HCl and Milli-Q water) and kept in holding tanks, cooled with flowing seawater, until further subsampling. To estimate Chl a, 500 mL of seawater collected in the previous step was subsampled and vacuum filtered (<100 mm Hg) through a 25 mm GF/F filter (Whatman, Florham Park, NJ, United States). The filtrate obtained was wrapped in aluminium foil and frozen for storage (−20°C) until further analysis. For nutrient analysis, filtrates of different depths were transferred to separate 100 mL PE bottles (pre-washed with 10% HCl) and stored frozen (−20°C). In addition, for nutrient analysis, filtrates of different depths were transferred to separate 100 ml PE bottles (pre-rinsed with 10% HCl) and stored frozen (−20°C). A detailed protocol for Chl a and nutrient estimation is given by Wu et al. (2019).

In order to calculate particle organic nitrogen (PON) and particulate organic carbon (POC), 300 ml of seawater samples were filtered onto 25 mm GF/F filters (pre-combusted at 450°C for 2 h) and kept frozen (−20°C) until further analysis. To eliminate inorganic carbon, the filters were fumigated with concentrated hydrochloric acid for 3 h prior to analysis. These fumigated filters were then examined with a Costech Elemental Combustion System (Costech Analytical Technologies Inc., Valencia, CA) according to established protocols (Solórzano and Sharp, 1980).

### DNA extraction, PCR and high-throughput sequencing

For DNA analysis, 500–800 ml of seawater samples from different sites was filtered separately under low vacuum (<100 mm Hg) using 0.22-μM GTTP filters (47 mm diameter, microporous, Eschborn, Germany). These filters were moved into 2 mL microtubes and quickly flash frozen in liquid nitrogen (−196°C) prior to analysis. The total DNA on filters was extracted using a DNasy PowerSoil Kit (Qiagen, Hilden, Germany) according to the manufacturer’s instructions. The quality of the DNA extracts was determined by 1.8% agarose gel electrophoresis (BioWest, Castropol, Spain). The concentration of DNA was also measured using a NanoDrop2000c spectrophotometer (Thermal Sciences, Wilmington, DE, United States). As a final step, the extracted DNA was diluted to a final concentration of 1 ng/l and stored at −20°C until amplification.

The 16S rRNA gene V3-V4 variable region was amplified using the common primers 343F (5′-TACGGRAGGCAGCAG-3′) and 798R (5′-AGGGTATCTAATCCT-3′). Diluted DNA was used as template for polymerase chain reactions (PCRs) using a Bio-Rad thermal cycler (Bio-Rad, Redmond, WA, United States). A detailed protocol for PCR and high-throughput sequencing is given in Text S1. High-throughput sequencing was performed at Shanghai OE Biotech CO., Ltd. (Shanghai, China) using the Illumina Miseq PE300 platform (Illumina, San Diego, CA, United States). Obtained raw sequencing data have been submitted to the NCBI Sequence Read Archive (SRA) under the following accession number: PRJNA613771. Raw sequencing data were saved in the FASTQ format, which includes details on raw sequences and the quality of the corresponding sequencing. Based on the corresponding barcode, the raw sequencing data were allowed to separate up to one mismatched sample (Zhang et al., 2017). The following bioinformatics analysis of the raw FASTQ files was performed using QIMME v1.8.0 (Caporaso et al., 2010), and the full protocol is included in Text S2. Finally, random resampling was performed in order to homogenize the sequences between samples.

### Detection of potential pathogen groups

Pathogen bacterial genera were identified from the virulence factor database (VFDB),^1^ which contains mainly human pathogens or including species endemic to the human gut. These taxonomic groups included *Aeromonas*, *Arcobacter*, *Campylobacter*, *Legionella*, *Acinetobacter*, *Coxiella*, et al. and members of the Enterobacteriaceae (e.g., *Yersinia*, *Klebsiella*, *Escherichia*, *Shigella*) (Liu et al., 2021). In addition, common foodborne (Scallan et al., 2011) and waterborne (Pond, 2013) human pathogens were included in our screening of 16S rRNA gene sequences, such as, *Listeria*, *Mycobacterium*, *Vibrio*, *Bacillus*, et al. The genera *Ochrobactrum*, *Porphyromonas*, *Rahnella* and *Pantoea* were also included in our screen as opportunistic pathogenic bacteria (Brady et al., 2008; Leight et al., 2018; Ryan and Pembroke, 2020; Xu et al., 2021).

### Statistical analysis

#### Alpha diversity analysis

To estimate the biodiversity of pathogenic bacterial communities, alpha diversity indices (Chao1 richness estimates, Ace richness estimates, Shannon diversity indices and Simpson diversity indices) have been measured based on OTU tables for each sample using R v4.0.5 software (R Foundation for Statistics Computing, Vienna, Austria). Statistical analysis of alpha diversity between different groups was determined by Kruskal–Wallis tests using Sigmaplot v14.0 software (Systat Software, Inc., United States). The result was visualized using box plots in OriginV8.5 software. Venn diagrams with shared and unique OTUs were utilized to depict the similarity and differences between different groups.

#### Beta diversity analysis

Beta diversity analysis mainly characterises the differences between biological communities. Beta diversity was calculated based on Bray-Curtis distances using the “Vegan” package of R v4.0.5. The monthly changes and depth differences in Bray-Curtis values between samples were then compared and shown using scatter plots in OriginV8.5. Non-metric multidimensional scaling (NMDS) analysis was performed using the ‘Vegan’ package of R v4.0.5 to reveal the spatial and temporal distribution patterns of pathogenic bacterial communities. NMDS results were visualized using the ‘ggplot2’ package of the R v4.0.5.

#### Co-occurrence network analysis

Networks analysis was used to reveal the interactions between bacterial communities. Spearman rank correlations between OTUs were calculated using the ‘Hmisc’ package of R v4.0.5 (Ju et al., 2014). The correlation between two OTUs was considered robust to subsequent network construction when the Spearman correlation coefficient |R| > 0.6 and value of *p* <0.05. In this process, network topology parameters were also calculated, including intermediate centrality, path length, proximity centrality, neighborhood connectivity distribution, pressure centrality distribution and shared neighborhood distribution. The analysis was visualized in Gephi v0.9.2 software. The statistical analyses were calculated using the “Vegetarian” and “Graph” packages of the R v4.0.5.

#### Multivariate statistical analysis

In order to analyze the relationship between pathogenic bacteria and the environmental factors, a multivariate statistical analysis was performed using the “Vegan” package of the R v4.0.5. The abundance of all observed pathogenic bacteria was used in this analysis. Firstly, a detrended correspondence analysis (DCA) was performed on all samples. The length of the longest gradient was less than 3, indicating that all samples were suitable for redundancy analysis (RDA). A total of 11 measured environmental parameters with VIFs<20 were used for further analyze. The relationship between environment (physicochemical factors), geographic distance, depth and beta diversity (Bray-Curtis distance) was revealed by Mantel and partial Mantel tests (with 999 alignments) (Legendre and Legendre, 2012). The relationship between each environmental parameter and the relative abundance of pathogenic bacteria was also detected by correlation test. Spearman correlation coefficients were calculated using SPSS (SPSS Inc., Chicago, Illinois, United States) software. The corresponding heat maps were plotted using the “ggplots” package of the R v4.0.5 (Warnes et al., 2005).

## Results

### Spatial variation of environmental characteristics

**Figure 2:**
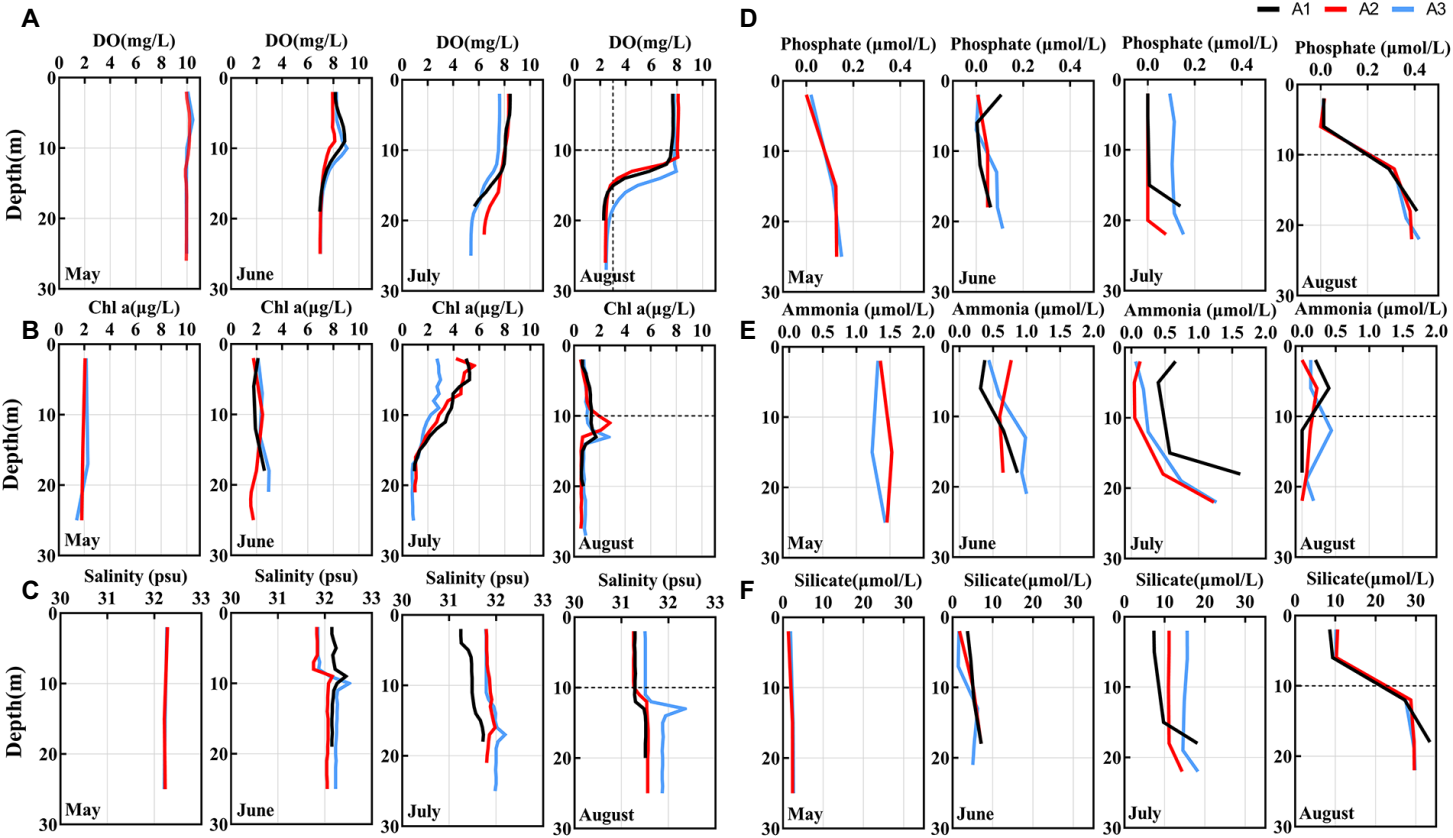
**(A–C)** Depth profiles of hydrological and chemical parameters from May to August in BHS. The hypoxia zone formed in August (The area below the dashed line of *Y*-axis).

The spatial and temporal variation in environmental characteristics of the study area is shown in Figure 2 and Supplementary Figure S1. From Supplementary Figure S1, seawater temperature in the surface waters of the study region gradually increases while the salinity decreases from May to August. In August, we observed that temperature decrease dramatically below 10 m, and the maximum temperature difference reached 8°C.

In May, the DO concentrations in the surface and bottom water were basically the same. From June, the DO concentrations gradually decreased, especially in the bottom water where the DO decreased more intensely (Figure 2A). Until August, the bottom DO concentrations reach a minimum value of 2.30 mg/L with a saturation of only 28.5%. The hypoxia zone developed to below 10 m where the DO concentration less than 3.01 mg/L, consistent with the location of low-temperature water. Similar to DO, the distribution pattern of pH also gradually decreased from May to August, and minimum pH values were observed below 10 m in August. Moreover, the pH in the hypoxia zone dropped below 7.8, reaching a summer minimum (Supplementary Figure S1).

However, Chl a concentration showed an opposite trend, gradually increasing from May, significantly increasing in July, and then decreasing in August (Figure 2B). Nutrient concentrations (phosphate, nitrate, nitrite, and silicate) gradually increased and accumulated in the hypoxia zone (< 10 m) in August (Figure 2 and Supplementary Figure S1). The concentration of ammonia is decreasing. Therefore, biological samples sampled at depths below 10 m were considered to be from bottom water (Bottom) while samples above 10 m (including 10 m) were from surface water (Surface). In August, for the analysis on a spatial scale, biological samples from depths below 10 m were also considered to be from the low oxygen (LO) environment while samples above 10 m (including 10 m) were from the high oxygen (HO) environment.

### Pathogenic bacteria diversity and richness

**Figure 3:**
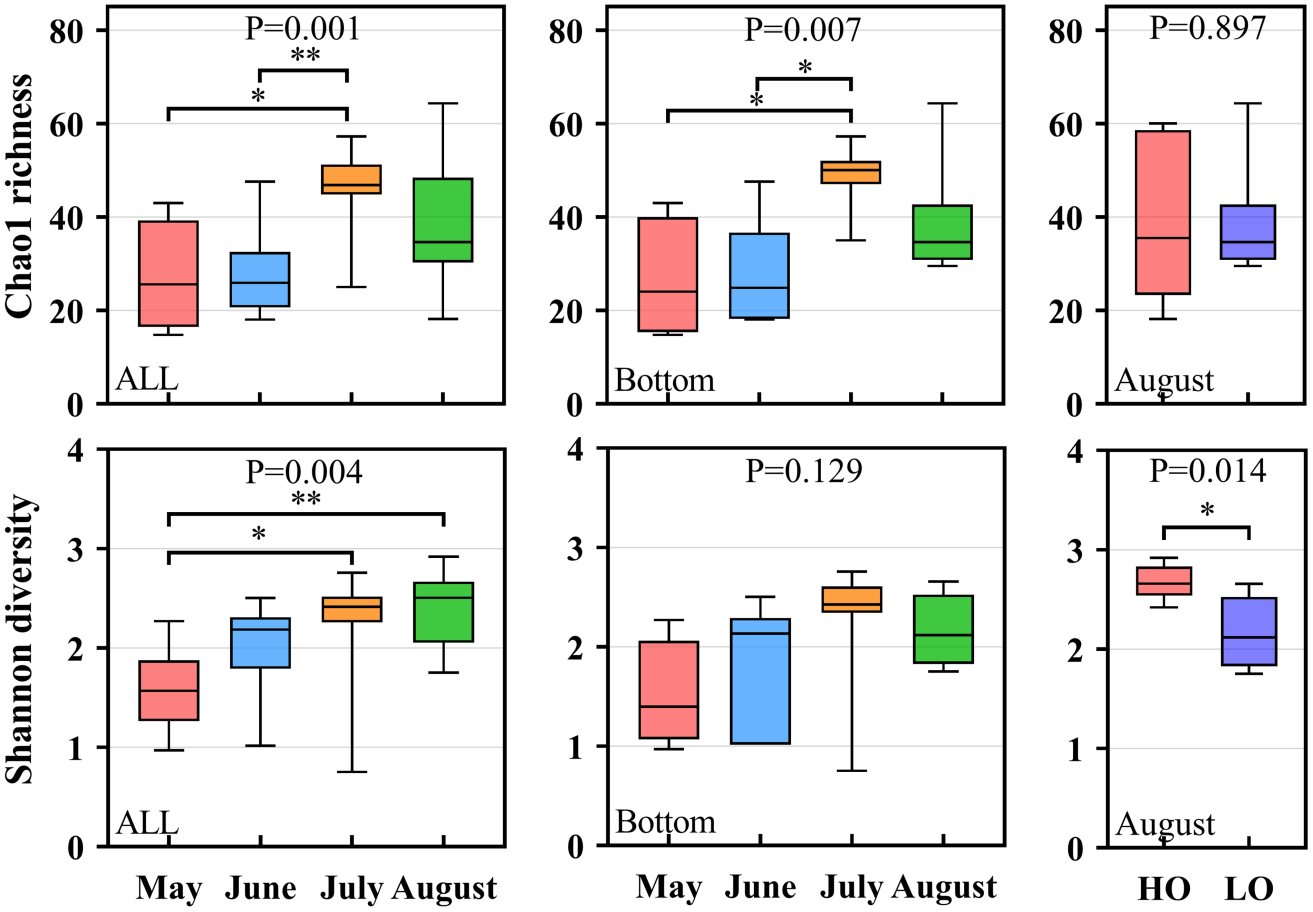
Box plots of alpha-diversity (Chao 1 index and Shannon Index) of the pathogenic bacterial communities in different groups in the Bohai Sea. *p < 0.05; **p < 0.001.

The 16S rRNA gene sequencing of these samples has been reported by previous study in our lab (Wu et al., 2022). According to previous report, a total of 3,043 OTUs were obtained in these samples of this region. Thus, the 3,043 OTUs were further used to detect pathogenic bacteria in our study. Finally, a total of 106 OTUs were identified as pathogenic bacteria in our study, which represent 2.99% of the total sequences. The richness index and diversity index of pathogenic bacteria were shown in Figure 3 and Supplementary Figure S2. From Figure 3, both the richness index and diversity index followed a temporal distribution. However, the Kruskal-Wallis test showed that the α-diversity index did not differ significantly (P>0.05) between sampling areas (Supplementary Figure S3). Overall, the Shannon index in the present study continuously increased from May to August, whereas Chao 1 index followed an inverted U-curve (Figure 3). The Chao 1 index showed that the richness was significantly higher in July than that in May and June, with a decrease in August (Figure 3). For the samples from the bottom water, the Shannon index increased from May to July but decreased in August. The Chao 1 index also had lower values in August (Figure 3). The Mann–Whitney test showed that Shannon, Simpson, and Pielou were significantly higher in the HO sample than in the LO sample (*p* < 0.05), indicating that some species could not tolerate the decrease of oxygen concentration in hypoxia zone of Bohai Sea.

**Figure 4:**
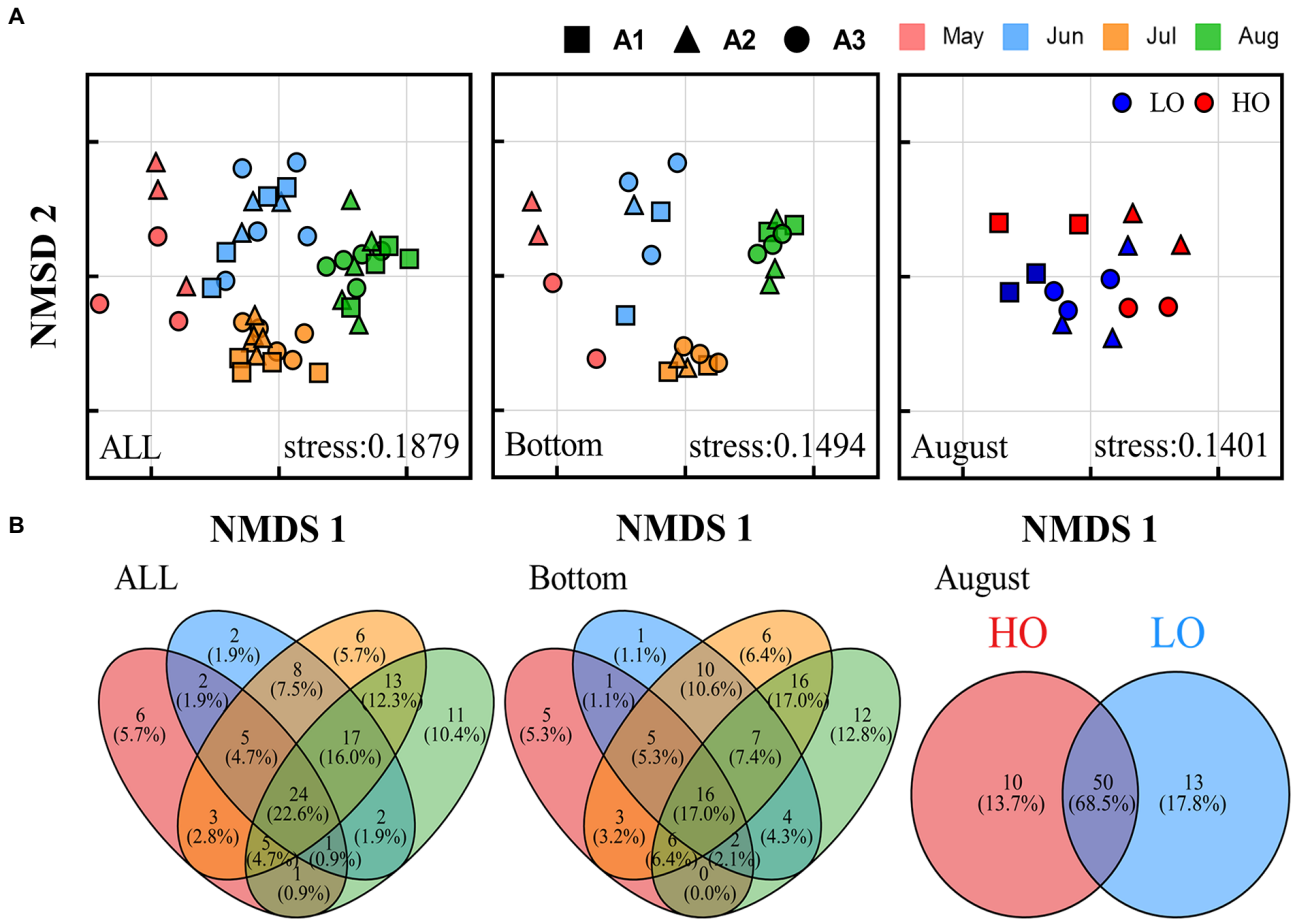
Community structure of pathogenic bacteria in the Bohai Sea over 4 months. **(A)** Non-metric multidimensional scale (NMDS) analysis of different microbial communities. **(B)** Venn diagram showing the number of unique and shared OTUs between different months for different communities.

Non-metric multidimensional scaling (NMDS) analysis based on Bray-Curtis distances showed that the pathogenic communities followed a significant temporal distribution pattern, with samples from the same month but in different regions clustered together, especially in the bottom water (<10 m) (Figure 4A). However, the distinction between regions was not clear. In August, LO and HO samples were not clearly separated and only part of LO samples were clustered together, suggesting that the effect of DO on the structural distribution of pathogenic bacteria communities may be limited.

**Figure 5:**
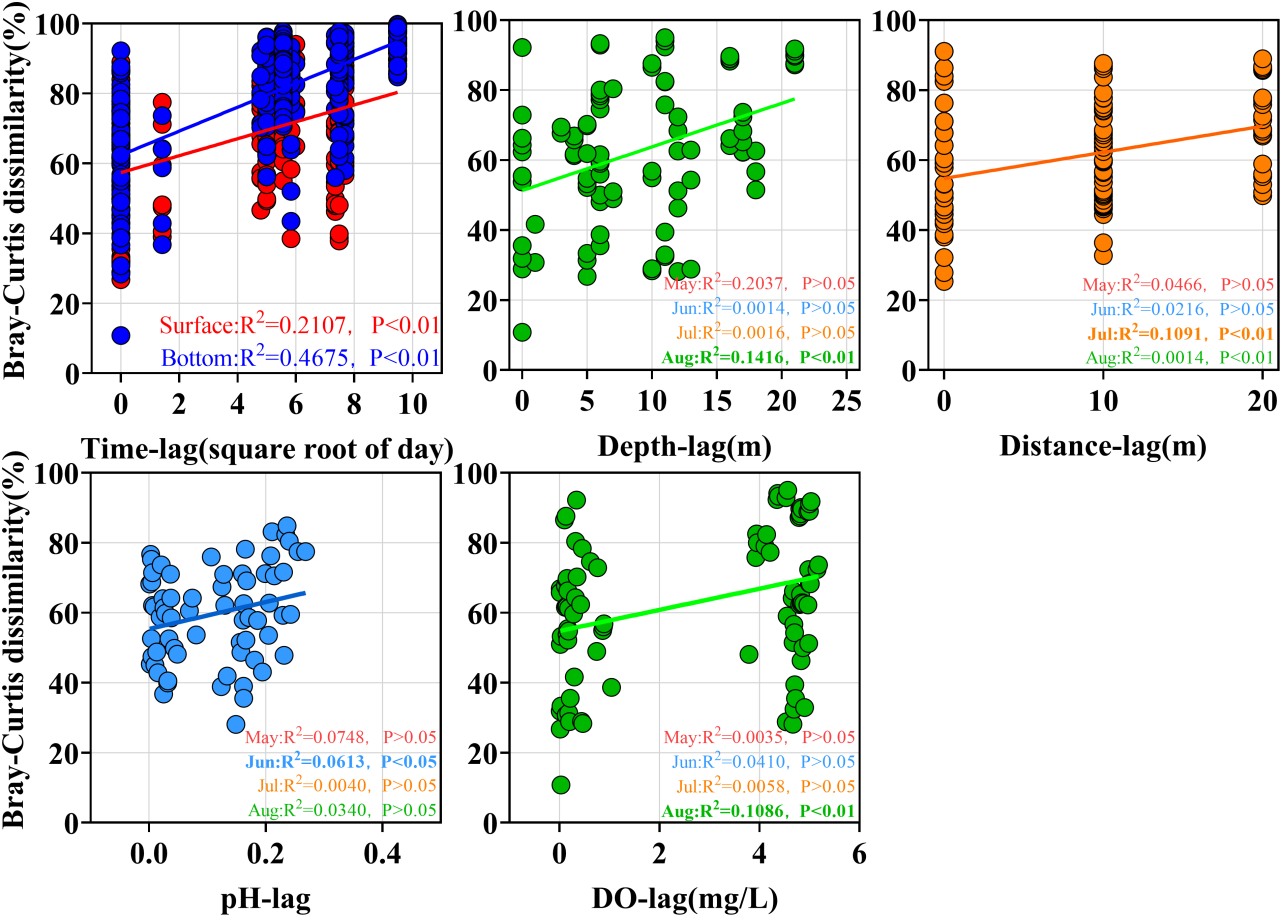
Regression analysis of Bray-Curtis differences for different pathogenic bacterial communities.

Venn diagram provides a visual representation of the variability and overlap in the OTU composition of pathogenic bacterial communities (Figure 4B). A total of 82 pathogenic OTUs (77.4%) changed over the 4 months, and this change was more pronounced in the bottom water (78 pathogenic OTUs, 83.0%). a large number of OTUs (68.5%) were shared between the HO and LO samples in August, and most species might not be sensitive to changes in dissolved oxygen. In addition, the time-lag regression analysis of Bray–Curtis dissimilarity all showed a positive slope (Figure 5). The slope of the Bottom was steeper than that of the Surface, indicating a directional change in the bacterial community. The changes of beta diversity in the bottom water accounted for a larger contribution. The depth-lag regression analysis of Bray–Curtis dissimilarity indicates that there is significant variability between samples from different depths in August. In July, geographical distance can be considered an important factor in shaping the variability of pathogenic bacterial communities. Similarly, there was significant variability between samples of different pH in June and DO in August.

### Community composition and distribution of pathogenic bacteria

**Figure 6:**
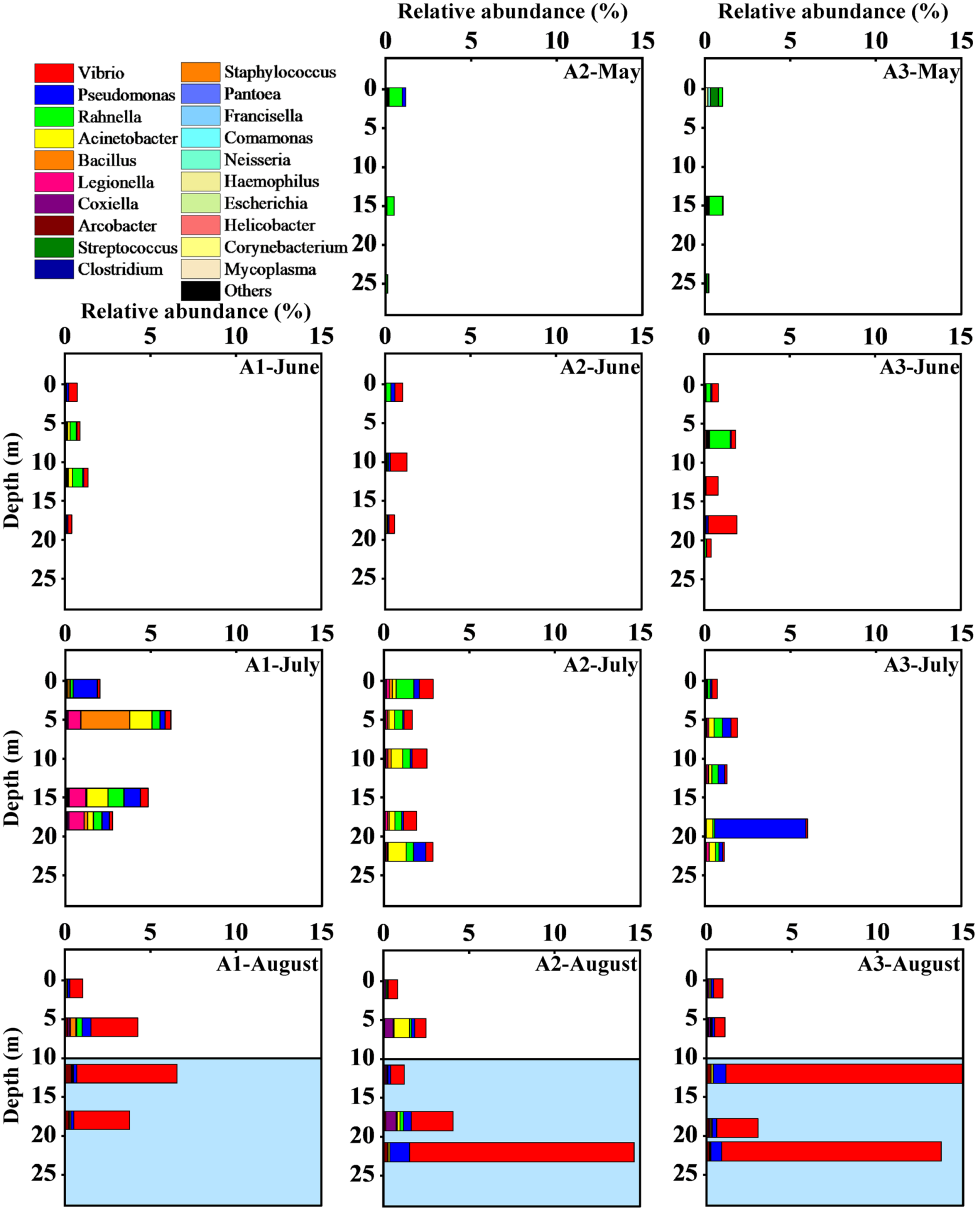
Vertical distribution of the relative abundance of pathogenic bacterial communities at the genus level from May to August.” Others” refers to OTUs with very low abundance (<0.001%). The hypoxia zone formed in August (The area marked in blue).

The 106 pathogenic bacteria OTUs belong to 5 phyla and 28 genera. From May to August, the number of pathogenic bacteria contributed 0.70, 1.00, 2.77, and 5.88% to the total number of bacteria (Figure 6). At the phylum level, the composition of the pathogenic community was similar for the 4 months, but with a difference in the percentage. Proteobacteria was dominated in all samples, accounting for 0.56, 0.96, 2.45, and 5.82% of the total bacteria in the 4 months. Firmicutes was the second dominant phylum with an increasing followed by decreasing trend in relative abundance, accounting for 0.13, 0.03, 0.31, and 0.05% of the total bacteria. Both Actinobacteria and Tenericutes accounted for less than 0.01% and did not change significantly. Bacteroidetes were present in only one sample, accounting for 0.004%.

Further analysis of the variation in the structure of pathogenic bacterial communities is discussed at the genus level. All 46 samples contained members of the genus *Pseudomonas* sp. and *Rahnella* sp., and most samples contained members of *Vibrio* sp., *Acinetobacter* sp., *Bacillus* sp., *Legionella* sp., *Coxiella* sp. and *Arcobacter* sp. Only three genera belonging to the Enterobacteriaceae were detected, including *Escherichia* spp., *Pantoea* sp. and *Rahnella* sp. *Campylobacter*, *Porphyromonas*, *Laribacter*, *Ochrobactrum*, *Moraxella*, *Aeromonas*, *Mycobacterium* and *Enterococcus* were detected in only a few samples with low relative abundance. In May, Streptococcus and *Rahnella* were the dominant taxa. *Acinetobacter*, *Rahnella*, *Pseudomonas*, and *Vibrio* had high relative abundance from June to August. In addition, *Legionella*, *Bacillus* and *Acinetobacter* showed higher values at the A1 station in July, *Arcobacter* and *Coxiella* cannot be ignored in August (Figure 6).

Some genera changed in the temporal gradient. *Vibrio* spp. showed the greatest variation in relative abundance, which was 0.001% in May and 5.01% in August, with insignificant changes between June and July (0.52 to 0.40%). The relative abundance of *Pseudomonas*, *Acinetobacter*, *Rahnella*, Escherichia, and Helicobacter showed an increasing trend from May to July and decreased in August, while *Coxiella* and *Corynebacterium* showed a continuously increasing trend. The relative abundance of *Arcobacter* increased significantly in August, while Clostridium decreased. *Legionella*, *Bacillus* and *Acinetobacter* had the same temporal trend, showing a high relative abundance in July and decreasing in August. In August, there is a clear vertical division of the pathogenic bacterial community between the hypoxia zone and the region with higher oxygen concentration (Figure 6). The Mann–Whitney test showed that the abundance of *Arcobacter* (*p* < 0.01), *Pseudomonas* (*p* < 0.05) and Vibrio (*p* < 0.01) in the LO was significantly higher than that in the HO (Supplementary Figure S4). In contrast, the abundance of *Rahnella* was higher in the high-oxygen region (*p* < 0.05; Supplementary Figure S4). Other genera showed no significant differences (*p* > 0.05, Supplementary Figure S4). Further statistical analysis revealed that the relative abundance of genera in the bottom water was more variable, a result that coincided with the time lag regression analysis of Bray–Curtis dissimilarity that Bottom exhibited a higher slope than the other groups (Figure 5).

### Co-occurrence network analysis

**Figure 7:**
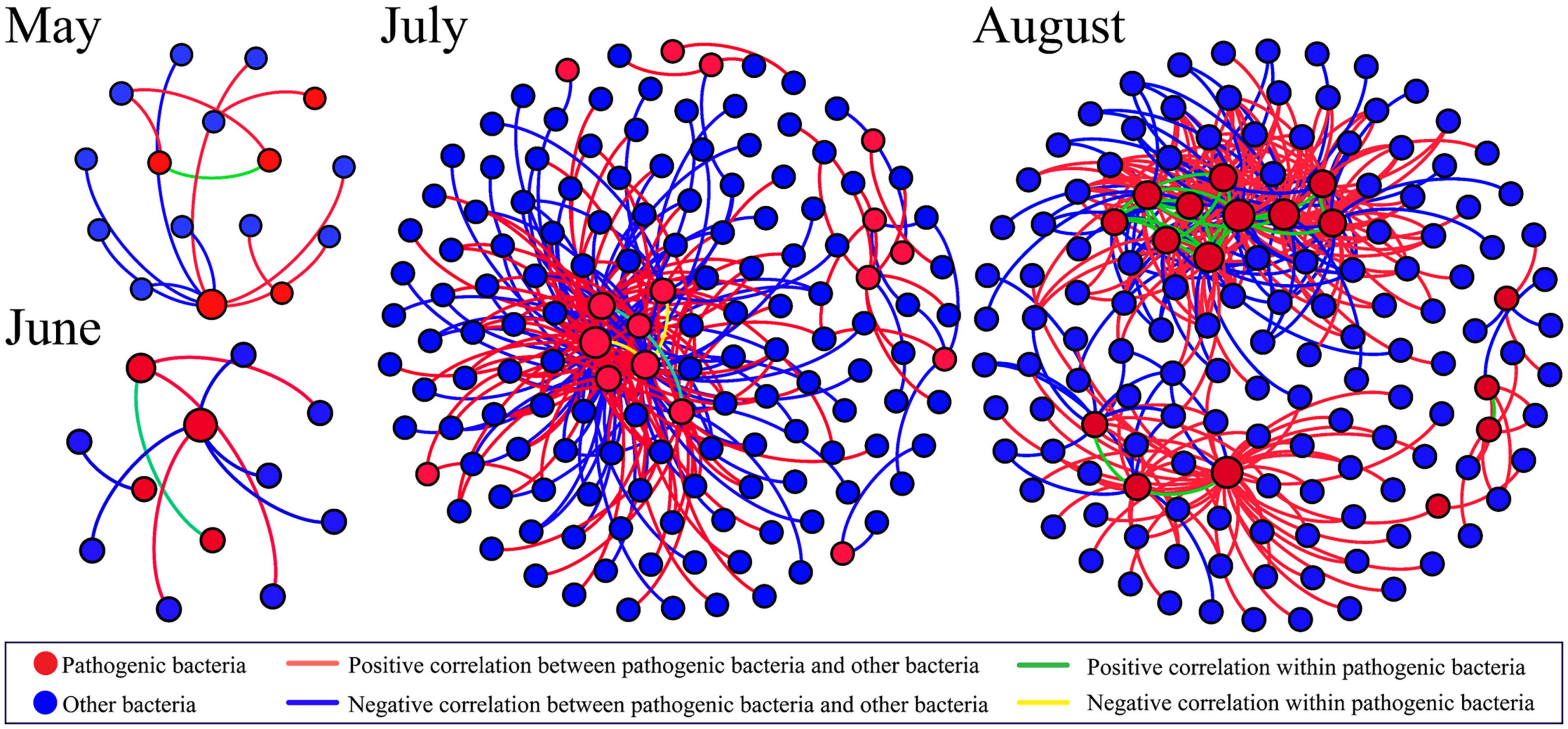
Network analyses showing the co-occurrence pattern within pathogenic bacteria and between pathogenic bacteria and other bacteria OTUs in the Bohai Sea from May to August. This figure shows significant correlations.

To investigate the network co-occurrence patterns in four different months, a co-occurrence network diagram (Figure 7) was constructed between the dominant pathogen and other bacteria OTUs (relative abundance >0.01%). The co-occurrence network preserves significantly related nodes as well as the linkages within pathogenic bacteria and between pathogenic bacteria and other bacteria. There were significant differences in the co-occurrence patterns of the networks for the four different months. The co-occurrence network in May retained 16 nodes (5 pathogenic OTUs) and 13 edges, while the co-occurrence network in June had 12 nodes (4 pathogenic OTUs) and 9 edges. Among them, OTU120 (*Rahnella*) had the highest degree in both months and was considered as the key taxon. In July, 146 nodes (17 pathogenic OTUs) and 232 edges were present. Although the network co-linear pattern was more complex, there was no significant change in the internal linkage of pathogenic bacteria. The number of positive correlations between pathogenic bacteria and other bacteria was higher than the number of negative correlations, indicating a greater facilitative than inhibitory effect between them. OTU120 (*Rahnella*), OUT1534 (*Leginoella*) andOTU318 (*Pseudomonas*) are the key taxa. Significantly, 148 nodes (16 pathogenic OTUs) and 348 edges were retained in the co-occurrence network in August, with the number of positive correlations much higher than that of negative correlations, indicating a closer ecological association between OTUs. Intra-pathogenic connections were significantly stronger (31 edges) and all were positively correlated, suggesting that the presence of hypoxia zones changes their symbiotic patterns and enhances the interactions between pathogenic bacteria. OTU133 (*Vibrio*), OTU318 (*Pseudomonas*) and OTU120 (*Rahnella*) were the key taxa.

### Relationships between pathogenic bacterial community structure and environmental parameters

**Figure 8:**
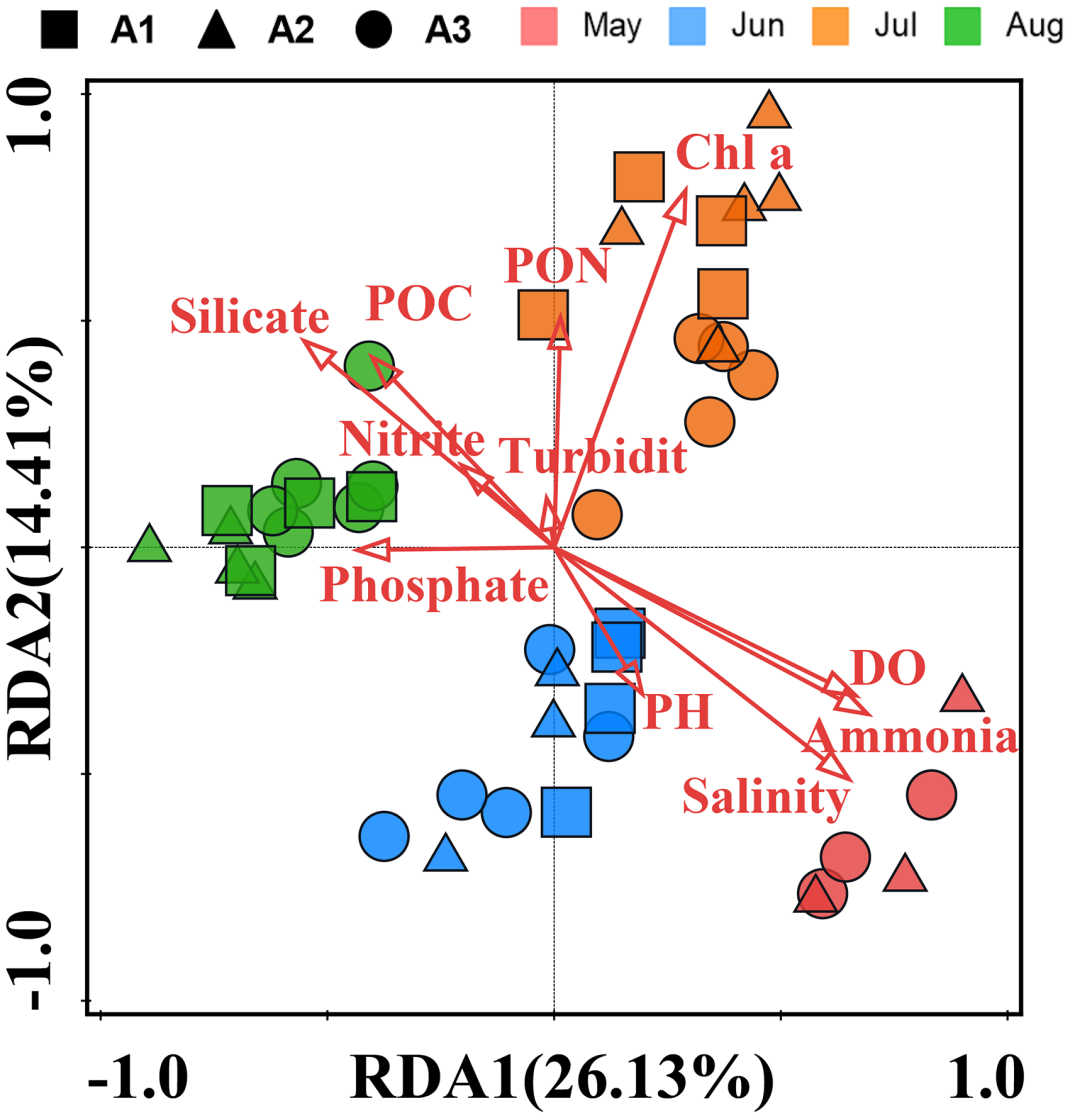
Redundancy analysis (RDA) was based on pathogenic bacteria composition and biotic/abiotic parameters as explanatory variables. The total variation explained in the abundance data is presented as two RDA axes (RDA1 and RDA2). Arrows represent environmental parameters. Different colored circles, boxes, and triangle symbols represent different samples.

The RDA analysis of samples with environmental parameters was performed (Figure 8). The environmental factors in the first two axis explained >40.54% of the total variance in the pathogenic bacteria community distributions. The distribution of samples in the RDA is similar to that of the NMDS, which also shows a clustering of samples from the same months. The RDA indicated that salinity, ammonia, DO, silicate, Chl a, POC, PON and phosphate were the main environmental parameters driving the distribution of pathogenic bacteria communities (*p* < 0.05), while nitrite, Turbidity and pH were not the main parameters (*p* > 0.05). Based on the 5% level in a partial Monte Carlo permutation test, Salinity (*p* = 0.001), Chl a (p = 0.001), DO (*p* = 0.002), silicate (*p* = 0.004), phosphate (*p* = 0.001) and ammonia (*p* = 0.007) contributed significantly to the total variance.

Mantel and partial Mantel tests further illustrated the relationship between pathogenic bacterial community structure and temporal factors, spatial factors (depth and geographic distance) and physicochemical variables (environment) in the study area (Table 1; Supplementary Table S1). The results showed that the β-diversity of 46 pathogenic bacterial communities was correlated with time and environment (*p* < 0.01, Table 2), with a weak distance effect (p > 0.05). The correlation between β diversity and environmental variables was not significant when controlling by time (p > 0.05; partial Mantel test). Thus, environmental variables were not considered the most important factor for overall pathogenic bacterial community β-diversity. Furthermore, the Mantel test revealed that in May and June, there was no significant association between β-diversity and known factors. Geographic distance influenced the β-diversity of pathogenic bacterial communities in July the most. The test also revealed that differences in pathogenic bacterial communities in August were connected to water depth and environment, with the partial Mantel test indicating that their correlations were possibly interdependent (|r| < 0.5, *p* > 0.05). Community differences were not significantly correlated with environmental parameters in May. For the pathogenic bacteria in June, community differences were associated with phosphate and nitrite. Salinity was a significant environmental factor affecting the beta diversity of the pathogenic community in July (*p* < 0.05, Mantel test in Supplementary Table S1), while nitrate and nitrite were controlled by geographical distance. In August, β diversity was influenced by DO, phosphate, nitrate and silicate. The correlation of DO, phosphate, nitrite and silicate with the diversity of β depended on the water depth.

**Table 1:** Topological properties of the co-occurrence network of pathogenic bacterial communities.

	CC	APL	ND	AD	GD
May	0.750	1.683	2	1.625	0.108
June	0.000	1.591	3	1.500	0.136
July	0.296	3.100	8	3.178	0.022
August	0.680	3.356	8	5.490	0.033

CC, clustering coefficient; APL, average path length; ND, network diameter; AD, average degree; GD, graph density.

**Table 2:** Mantel and Partial Mantel tested Spearman correlations between geographic distance, collection depth, environment, and beta diversity.

Samples	Variables	Mantel tests	Partial mantel tests control factors			
			**Time**	**Depth**	**Environment**	**Distance**
All	Time	**0.663**	\	**0.663**	**0.629**	**0.665**
	Depth	0.019	−0.001	\	−0.091	0.020
	Environment	**0.288**	−0.103	**0.192**	\	**0.288**
	Distance	0.017	0.010	−0.013	0.027	\
July	Depth	0.126	\	\	0.019	0.169
	Environment	−0.012	\	−0.136	\	−0.041
	Distance	**0.341**	\	**0.358**	**0.340**	\
August	Depth	**0.390**	\	\	0.248	**0.390**
	Environment	**0.310**	\	−0.004	\	**0.309**
	Distance	−0.012	\	0.029	0.003	\

Tests were performed using all samples, and samples from different months, respectively. Correlations are shown in this table. Bold correlations were corrected by Benjamini-Hochberg multiple tests when the value of *p* was less than 0.05. The environment was calculated as the Euclidean distance of the physicochemical parameters.

**Figure 9:**
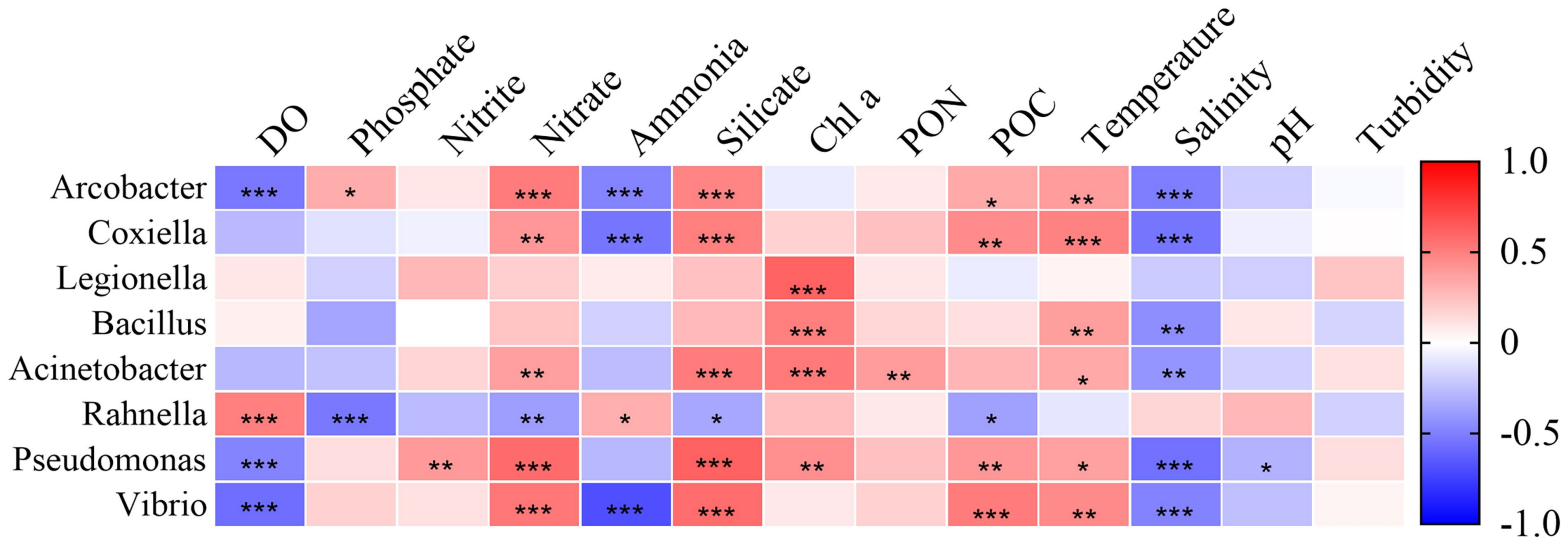
Environmental factors associated with changes in the community structure of pathogenic bacteria at the genus level.

The correlation between the relative abundance of species and environmental factors at the genus level was investigated by Spearman-Heatmap correlation analysis (Figure 9). The top eight genera in relative abundance were selected as representatives. The results found that many environmental parameters including temperature, salinity, DO, nutrients (nitrate, ammonia and phosphate) and silicate play an important role in species composition, while turbidity is not major influencing factors. The main genus with significant positive correlation with temperature was *Arcobacter*, *Coxiella*, *Bacillus*, *Acinetobacter*, *Pseudomonas* and *Vibrio*. Similarly, this genus had a significant negative correlation with salinity. DO have a significant negative correlation with *Arcobacter*, *Pseudomonas* and *Vibrio*. *Rahnella*, however, had a significant positive correlation with DO. pH and nitrite were significantly correlated with *Pseudomonas* only. Nitrate, silicate and POC had significant positive correlations with *Arcobacter*, *Coxiella*, *Acinetobacter*, *Pseudomonas* and *Vibrio*. The species with negative correlation with ammonia were *Arcobacter*, *Coxiella* and *Vibrio*. in contrast to other genus, *Rahnella* had negative correlation with phosphate, nitrate, silicate and POC, and positive correlation with ammonia. In addition, *Legionella*, *Acinetobacter* and *Bacillus* had a significant positive correlation with Chl a.

## Discussion

### Spatial and temporal distribution of pathogenic bacteria

In this study, deep amplification sequencing of the 16S rRNA gene was performed and many bacterial families and genera containing pathogenic bacteria were identified to be present in the study area from May to August. The pathogenic bacterial community in the study region was mainly consisted of the *Proteobacteria* and *Firmicutes*, and Proteobacteria was dominant group in all 4 months. Our study identified *Vibrio*, *Pseudomonas*, *Rahnella*, *Acinetobacter*, *Bacillus*, *Legionella*, *Coxiella* and *Arcobacter* as the dominant pathogenic bacteria. Similar dominant taxa composition has also been observed in other marine ecosystems (Leight et al., 2018; Xu et al., 2018). The ubiquity of some groups, such as *Vibrio*, *Pseudomonas* and *Bacillus*, is well-known (Wang et al., 2019). The risk to human health from other low abundance pathogenic genera should not be ignored.

All the dominant genera changed in a temporal gradient. Among them the relative abundance of *Vibrio*, *Pseudomonas*, *Rahnella*, *Acinetobacter* and *Coxiella* showed an increasing trend from May to July. Similar patterns have been found in other study seas (Crump et al., 2004; Comte and del Giorgio, 2010; Pereira et al., 2015), temporal variation of bacterial communities is correlated with environmental factors. Most of the dominant pathogenic bacteria showed a strong correlation with temperature, salinity, and nutrients (Figure 9). Strong sunlight, nutrient enrichment and warmer water temperatures in summer may have accelerated the rate of cellular metabolism, causing a rapid increase in bacterial abundance (Cochran and Paul, 1998). Mantel tests suggest that the strong association of temperature and salinity on pathogenic bacteria might base on temporal rather than spatial scales (Supplementary Table S1). Notably, *Legionella*, *Bacillus* and *Acinetobacter* were present at high levels at station A1 in July, while the other genera did not differ significantly in geographic distance. This variation followed the dynamics of Chl a (Figure 9). In general, Chl a reach their highest levels of the year in summer due to the large amount of nutrients carried by surrounding terrestrial runoff into the BHS and sufficient sunlight (Sun et al., 2012). As a common native taxonomic group of freshwater and estuarine waters (Grimes, 1991; Nemec et al., 2010), *Legionella*, *Bacillus* and *Acinetobacter* may be derived from terrestrial recharge due to continuous precipitation. The Spearman correlation test showed (Figure 9) that most environmental parameters were not significantly correlated with *Legionella*, *Bacillus* and *Acinetobacter*. This is further evidence that they may come from exogenous inputs. In addition, as pathogens, *Legionella* often causes Legionella pneumonia, which can be life-threatening (Talapko et al., 2022). *Bacillus* and *Acinetobacter* are mostly opportunistic bacterial pathogens that cause a variety of symptoms after infection (Johnson et al., 2015).

In August, the relative abundance of *Vibrio*, *Coxiella* and *Arcobacter* increased significantly, with *Vibrio* and *Arcobacter* showed a clear division in the vertical direction (Figure 6). The Mann–Whitney test showed significantly higher abundances of *Vibrio* (*p* < 0.01) and *Arcobacter* (*p* < 0.01) in the hypoxia zone than in the surface water (Supplementary Figure S4). This variation may be dominated by DO and nutrient components (Figure 9). Most species of *Vibrio* and *Arcobacter* are facultative anaerobe and higher oxygen concentrations can restrain their growth (Johnson et al., 2012; Mudadu et al., 2021). Conversely, eutrophic and anoxic conditions in the hypoxia zone favor their proliferation (Moncada et al., 2019). And the highly variable traits and life cycles of *Vibrio* sp. (Thompson et al., 2004) lead to an absolute dominance of their relative abundance. In contrast, *Coxiella* showed no sensitivity to hypoxia and no obvious differences in spatial distribution, which may be related to his adaptability. In addition, *Vibrio* is a common bacterium of seawater, most species of which are pathogenic and often cause food poisoning and acute diarrhea (Wang et al., 2019). *Coxiella* is the causative agent of Q fever. It is a zoonotic disease that can be contracted by humans through inhalation of contaminated aerosols (Beare et al., 2009). *Arcobacter*, as a zoonotic food- and water-borne pathogen is closely associated with diarrhea and bacteremia in humans and animals (Ho et al., 2006). The increased abundance of pathogenic bacteria, which greatly increases the risk of disease, is of great concern.

Meanwhile, the relative abundance of *Pseudomonas* and *Rahnella* showed a decreasing trend in August, but still remained at a high level. The Mann–Whitney test showed that the abundance of *Rahnella* was higher in the hypoxia zone (*p* < 0.05; Supplementary Figure S4), while *Pseudomonas* was the opposite. *Pseudomonas* is ubiquitous in the environment and contains clinically important human pathogens such as *P. aeruginosa*, *P. putida* and other opportunistic pathogens (Zhu et al., 2013). *Rahnella* as a conditional pathogen could infect humans, especially those who are immunocompromised or suffer from underlying chronic diseases. Our study indicated that changes in *Pseudomonas* were associated with Chl a (Figure 9), and the decrease of primary productivity in August suggests that organic carbon limitation might be the main reason for the decrease in *Pseudomonas*. However, many *Pseudomonas* species have an intact denitrification pathway (Zhan et al., 2016), and these species might have higher adaptations in the hypoxic zone and maintained higher levels. Previous studies have shown a preference for polymer organic phosphorus of *Rahnella* (Zhao et al., 2019), which may be better adapted to the metabolism of macromolecular organic matter. This represents an environment where DO has not yet begun to run out. So when the DO decreased and the organic matter degraded, these microbes were gradually replaced in the environment by bacteria with a greater preference for small-molecule organic matter.

In summary, pathogenic bacteria were commonly distributed throughout the sampling sites (Figure 6) and varied over time. The central Bohai Sea was threatened by different pathogenic bacterial groups at different times. The dominant pathogenic taxa were strongly influenced by environmental conditions, consistent with their physiological requirements and ecological functions. Changes in temperature and salinity as well as DO and nutrient salt nutrient (nitrate, phosphate, silicate) concentrations had significant effects on the abundance distribution of dominant pathogenic bacteria (Figure 9). This also indicates that the specific ecology of the hypoxic led a greater variation in the relative abundance of the dominant pathogenic bacteria. The influence of nutrients on the distribution of dominant genera has exceeded that of DO. Half of the dominant genera were not significantly correlated with DO, although most of them were considered as exotic taxa. Nevertheless, DO still had a significant effect on the spatial distribution pattern of pathogenic genera. Our study found that the abundance of *Vibrio* and *Arcobacter* in the hypoxia zone increased significantly, and the abundance of *Pseudomonas* was maintained at a high level. And the hypoxic may keep developing in the coming decades (Zhai et al., 2019), which may keep enhancing the healthiness threat posed by *Vibrio*, *Arcobacter*, and *Pseudomonas*.

### Different factors shaped the community structure of pathogenic bacteria

In the present study, pathogenic bacterial communities varied significantly over time. Spatial factors (depth and geographic distance) and environment also contributed to the diversity of bacterial communities, and their influence varied in different months (Table 2). With increasing time delay, the Bray–Curtis dissimilarity between samples greatly increased (Figure 5), indicating that the pathogenic community had reached another state (Xue et al., 2018). Our results showed that the time lag regression slope of the samples in the bottom water (*R*^2^ = 0.4675, *p* < 0.01) was greater than that of surface water (*R*^2^ = 0.2107, *p* < 0.01), suggesting that members of the bottom water may have undergone more drastic changes, which is consistent with the results of the Venn diagram. Although depth and geographic distance are commonly thought to be important factors influencing the distribution of planktonic bacterial communities (Stevens and Ulloa, 2008; Zheng et al., 2016), there was no significant effect observed in May to June. β-diversity of pathogenic bacteria in July is influenced by geographical distance. And for August, while the Mantel test indicated that pathogenic β-diversity was correlated with depth and environment, the partial Mantel test suggested that their correlations may be interdependent. This emphasizes that temporal differences in pathogenic bacterial composition overwhelm spatial differences and may be influenced by changing environmental factors (Xue et al., 2018; Chen and Zhang, 2019). Environmental parameters such as TP, pH, and DO, which are regarded to be key drivers of community composition changes in marine settings, are constantly related to bacterial community structure and dispersion (Yu et al., 2018). Our findings revealed that salinity, ammonia, DO, phosphate, silicate, and Chl a were the key environmental variables responsible for changes in pathogenic bacteria community structure according to RDA analysis. However, the effects of pH on the pathogenic bacterial community were not significant. The shift in salinity is the most visible alteration in the marine environment. Water masses with different salinities showed varied planktonic bacterial communities (Zheng et al., 2016), and similar findings have been seen in other seas (Lozupone and Knight, 2007), suggesting that salinity is a key component in controlling pathogenic bacterial communities. DO was clearly correlated with pathogenic bacterial community structure, although there was overlap between samples from high and low oxygen concentrations (Figure 4). Oxygen concentration was previously considered as one of the main driving forces in shaping microbial communities in water (Liu et al., 2015) Similarly, many other environmental parameters including nutrients (nitrate, ammonia and phosphate) showed a significant correlation with pathogenic bacterial communities. This emphasizes that nutrients might play a crucial role in the formation of bacterial communities (Mo et al., 2018). As previously observed, the biogeography of bacterial communities in the research region was significantly impacted by silicate as a primary limiting factor (Zhang et al., 2014). This might be because the growth of bacterial communities depends on the large amount of DOC released by the flourishing diatoms, and silicate becomes vital as a necessary nutrient for the growth of various diatoms (Töpper et al., 2010). In addition, temperature and nitrate were not included in our RDA analysis due to high covariance with other factors. However, this does not eliminate the potential role of these factors in the construction of pathogenic community heterogeneity. Previous studies have also shown that the effect of temperature on total bacterial diversity and growth in the natural environment is significant (Kleinteich et al., 2012). In addition, many studies have confirmed that elevated temperatures may enhance pathogen virulence and growth (Maurelli, 1989; Vojvodic et al., 2011).

Environmental factors causing differences in pathogen community structure between samples were variable over 4 months (Supplementary Table S1). In May, the environmental variables detected did not have a significant effect on the pathogenic bacterial community, which could be determined by other biotic and abiotic factors. In addition to physical and chemical variables, phytoplankton composition, grazing and viral infection play an important role in the formation of bacterial community structure (Beardsley et al., 2003; Pinhassi et al., 2004; Grossart et al., 2005; Bouvier and del Giorgio, 2007; Weinbauer et al., 2007). In June, nitrite and phosphate significantly affected pathogenic community composition. in July, pathogenic community structure was influenced by nitrate, nitrite and salinity, and all showed dependence on geographic distance. It is suggested that seasonal heterogeneity in central Bohai Bay, such as seasonal hydrodynamic conditions and riverine input, may be responsible for the differences in pathogenic bacterial community structure. In August, coastal stratification created unique environmental conditions in the study area. This is mainly reflected in the significant decrease of oxygen concentration and pH below 10 m and the accumulation of nutrients (nitrate, phosphate, and silicate). The relationship between the depletion of DO, the decrease in pH and the corresponding enrichment of DIC suggests that aerobic respiration of organic matter is the main cause of oxygen depletion in the hypoxia zone and causes short-term acidification (Zhao et al., 2017). Stratification is necessary for the formation and maintenance of hypoxia (Irby et al., 2016). Anoxic conditions also inhibit biological activity thereby reducing the rate of remineralization of organic matter, which can result in the accumulation of organic matter in the hypoxia zone (Jessen et al., 2017). Our study observed that pathogenic bacterial communities are vertically stratified in marine hypoxia systems. This is consistent with previous studies (Stevens and Ulloa, 2008). Diversity of pathogenic bacteria was significantly reduced in the hypoxia zone. As other bacteria, the vertical stratification of pathogenic bacterial communities appears to be associated with specific characteristics of Hypoxia. The Mantel test indicated that the β-diversity of pathogenic bacteria was correlated with the concentration of DO, phosphate, nitrate and silicate, while partial Mantel tests suggested their correlations were controlled by depth (Supplementary Table S1). Depth is a composite indicator of many physicochemical factors as previously reported (Fortunato et al., 2011; Li et al., 2014; Gong et al., 2015). However, the feedback to changes in DO vary considerably among species, with some species being sensitive to changes in DO. but most species were not, and samples of HO and LO still shared a large number of OTUs (Figure 4B).

### Co-occurrence pattern of pathogenic bacteria in coastal hypoxia zone

The co-occurrence network showed that the pathogenic networks also differed between months. In May and June, the network topology was simple and not strongly connected. The co-occurrence network got more complicated in July, as the diversity of pathogenic bacteria expanded, with greater and predominantly positive linkages between pathogenic bacteria and other bacteria, but relative independence amongst pathogenic bacteria. In August, the co-occurrence network includes more nodes and linkages, indicating greater connectivity between species in the community, particularly pathogenic bacteria. The correlations of microbial communities’ change depending on their nutrient preferences and ecological niches (Banerjee et al., 2016). The increase in the total amount and variety of nutrients in the hypoxia zone creates more ecological niches, which promotes co-use among species and, as a result, more cooperative interactions. This indicates that in July and August, complex networks will result in increased community stability and resilience to disruptions (Yang et al., 2019). All of the networks in this study exhibited positive rather than negative interactions, indicating that microbial cooperation rather than competition is more prevalent. The important nodes found in this study all belonged to Proteobacteria, and it appears that Proteobacteria controlled these interactions. It’s worth noting that the functions of several nodes in these four networks remained unchanged. OTU120 (*Rahnella*) in 4 months, OTU318 (*Pseudomonas*) in July and August, implying that particular microbial functions are unaffected by environmental impacts or disturbances (Ling et al., 2016; Yang et al., 2019).

## Conclusion

In this study, through continuous sampling efforts from May to August 2017, we observed significant differences in environmental conditions along a temporal gradient, with increased hypoxia detected below 10 m north of the binuclear structure in the region. We detected pathogenic bacteria at all sites by high-throughput sequencing of 16S rRNA genes, which was often ignored before. Pathogenic bacterial communities are highly temporally heterogeneous and regulated by environmental factors. Depth and geographic distance had less influence over temporal gradients. The community in the bottom water had a stronger variation on the temporal gradient. A significant separation of pathogenic bacterial communities in surface water and hypoxia zone was observed in August. This can be explained by the stratification of the water column and changes in a variety of environmental factors such as DO and nutrients. However, the pathogenic bacteria community structure is not sensitive to changes in pH. In conclusion, the study area is exposed to the threat of changing pathogenic bacterial communities and community stability is increasing from May to August. The occurrence of hypoxia significantly increases the prevalence of pathogenic bacteria, most notably *Vibrio* and *Arcobacter*. The developing hypoxia zone may increase this phenomenon which poses a serious threat to human health. This study used 16S rRNA high-throughput sequencing to provide useful information on the abundance, distribution and temporal dynamics of pathogenic bacteria occurring in the central Bohai Sea, and to inform decisions regarding the safety of the sea area.

## Data availability statement

The data presented in the study are deposited in the NCBI Sequence Read Archive (SRA), accession number PRJNA613771.

## Author contributions

YG and CW: Methodology. JS: Conceptualization, resources, supervision, project management and access to funding. YG: writing - original manuscript preparation, data curation, formal analysis, software, and visualisation. CW and JS: writing - review and editing. All authors: read and agree to the published version of the manuscript.

## Funding

This work was supported by the National Key Research and Development Project of China (No. 2019YFC1407800), National Natural Science Foundation of China (No. 41876134), and Changjiang Scholar Program of Chinese Ministry of Education (No. T2014253) to JS. This research was also financially supported by State Key Laboratory of Biogeology and Environmental Geology, China University of Geosciences (Nos. GKZ21Y645 and GKZ22Y656).

## Conflict of interest

The authors declare that the research was conducted in the absence of any commercial or financial relationships that could be construed as a potential conflict of interest.

## Publisher’s note

All claims expressed in this article are solely those of the authors and do not necessarily represent those of their affiliated organizations, or those of the publisher, the editors and the reviewers. Any product that may be evaluated in this article, or claim that may be made by its manufacturer, is not guaranteed or endorsed by the publisher.

## Footnotes

^1^ http://www.mgc.ac.cn/VFs/
