# Supplementary material for: Pathogenic bacteria significantly increased under oxygen depletion in coastal waters: A continuous observation in the central Bohai Sea
Source: Front Microbiol. 2022 Nov 21;13:1035904. doi: 10.3389/fmicb.2022.1035904 (PMC9719909; doi:10.3389/fmicb.2022.1035904)
Supplement: Supplementary file 1 [file Data_Sheet_1.docx]

Pathogenic bacteria significantly increased under oxygen depletion in coastal waters: a continuous observation in the central Bohai Sea

**Yiyan Guo^1^, Chao Wu^2, 3^, Jun Sun^1, 4*^**

^1.^Research Centre for Indian Ocean Ecosystem, Tianjin University of Science and Technology, Tianjin 300457, China

^2.^Key Laboratory of Sustainable Development of Marine Fisheries, Ministry of Agriculture and Rural Affairs, Yellow Sea Fisheries Research Institute, Chinese Academy of Fishery Sciences, Qingdao, China

^3^ Laboratory for Marine Fisheries Science and Food Production Processes, Pilot National Laboratory for Marine Science and Technology, Qingdao, China

^4^ College of Marine Science and Technology, China University of Geosciences (Wuhan), Wuhan 430074, China

***Correspondence:**JunSun
E-mail: [phytoplankton@163.com](mailto:phytoplankton@163.com)

**Text S1 Detailed protocol of PCRs and high throughput sequencing**

A detailed PCR method is provided by Lv et al. (2018). By using 1.8% agarose gel electrophoresis to check the integrity of the amplified DNA, the product with a band of approximately 465 bp was considered to have efficient amplification. Following that, the PCR products were purified using AMpure XP beads (Agencourt Bioscience, Beverly, MA, USA). A second round of PCR was performed in order to label the double-indexed barcodes and to isolate the samples after sequencing. the PCR products were again purified using AMpure XP beads and quantified by Qubit dsDNA assay kit (Thermal Scientific, Wilmington, DE, USA). Finally, equal amounts of purified PCR products were pooled for subsequent sequencing.

**Reference**

Lv, Z., Wang, J., Yang, G., Feng, L., Mu, J., Zhu, L. and Xu, X. (2018). Underestimated effects of sediments on enhanced startup performance of biofilm systems for polluted source water pretreatment. *Biodegradation*, [online] 29(1), pp.89–103. doi:10.1007/s1053201798158.

**Text S2 Quality control and sequencing data processing**

Ambiguous bases were firstly detected and cut off from the paired-end reads via Trimmomatic v0.33 software (Bolger et al., 2014). Moreover, low-quality sequences (avg. Phred score <20) were also discarded in this software using a sliding window trimming approach. After quality control, the raw sequencing reads were assembled to full-length sequences through FLASH v1.2.7 software (Magoc and Salzberg, 2011). The parameters used in the assembly steps were set as follows: minimal and maximum overlapping length is 10 and 200 bp respectively, maximum mismatch rate = 20%. The emerged full-length sequences were further denoised to get high-quality sequences including removal of sequences less than 200 bases, ambiguous base containing sequences, and homopolymer containing sequences. Chimera sequences were subsequently detected from the quality-filtered sequences with UCHIME v4.2 software and removed before downstream analysis (Edgar et al., 2011). Then, the barcodes, linker sequences, and primers were removed from the remaining effective sequence to generate the final effective tags. After quality filtered, the remaining effective tags were clustered at a 97% similarity cutoff to generate operational taxonomic units (OTUs) using the open-source pipeline VSEARCH (Torbjørn et al., 2016). The most common sequences were selected as the representative sequences of each OTU in the QIMME pipeline (Quast et al., 2012). All representative sequences were aligned, annotated, and blasted against SILVA v123 through the RDP classifier. To clarify, the confidence threshold used in the process of annotation was set as 70%.

**Reference**

Bolger, A.M., Lohse, M. and Usadel, B. (2014). Trimmomatic: a flexible trimmer for Illumina sequence data. Bioinformatics, 30(15), pp.2114–2120. doi:10.1093/bioinformatics/btu170.

Magoc, T. and Salzberg, S.L. (2011). FLASH: fast length adjustment of short reads to improve genome assemblies. Bioinformatics, 27(21), pp.2957–2963. doi:10.1093/bioinformatics/btr507.

Edgar, R.C., Haas, B.J., Clemente, J.C., Quince, C. and Knight, R. (2011). UCHIME improves sensitivity and speed of chimera detection. Bioinformatics, [online] 27, pp.2194–2200. doi:10.1093/bioinformatics/btr381.

Torbjørn, R., Tomáš, Fl., Nichols, B., Quince, C., Mahé, F., 2016. VSEARCH: A versatile open-source tool for metagenomics. PeerJ 4, e2584. https://doi.org/10.7717/peerj.2584

Quast, C., Pruesse, E., Yilmaz, P., Gerken, J., Schweer, T., Yarza, P., Peplies, J. and Glöckner, F.O. (2012). The SILVA ribosomal RNA gene database project: improved data processing and web-based tools. Nucleic Acids Research, [online] 41(D1), pp.D590–D596. doi:10.1093/nar/gks1219.


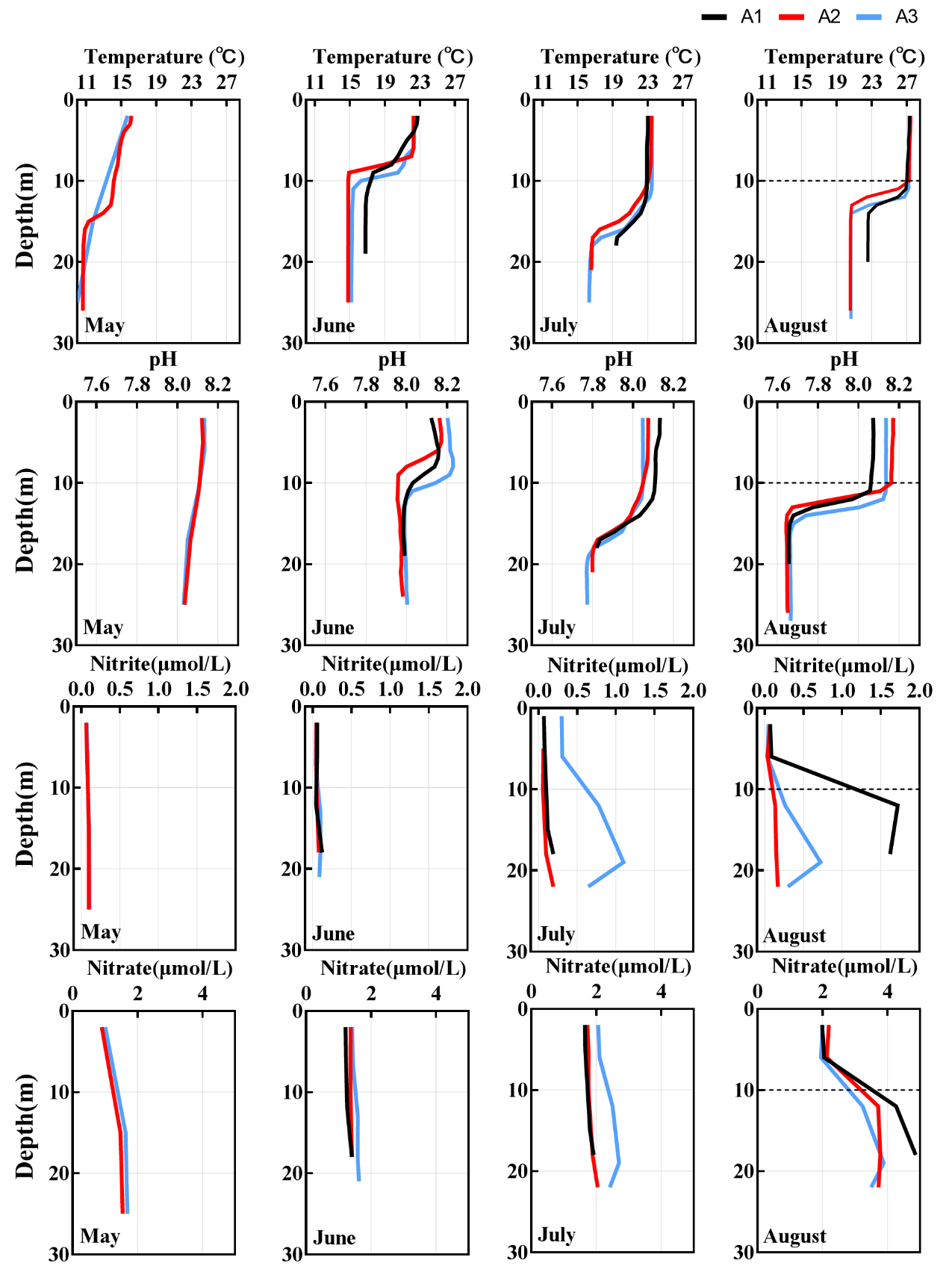


**Fig. S1** Depth profiles of temperature, pH, nitrite and nitrate in four months from May to August.


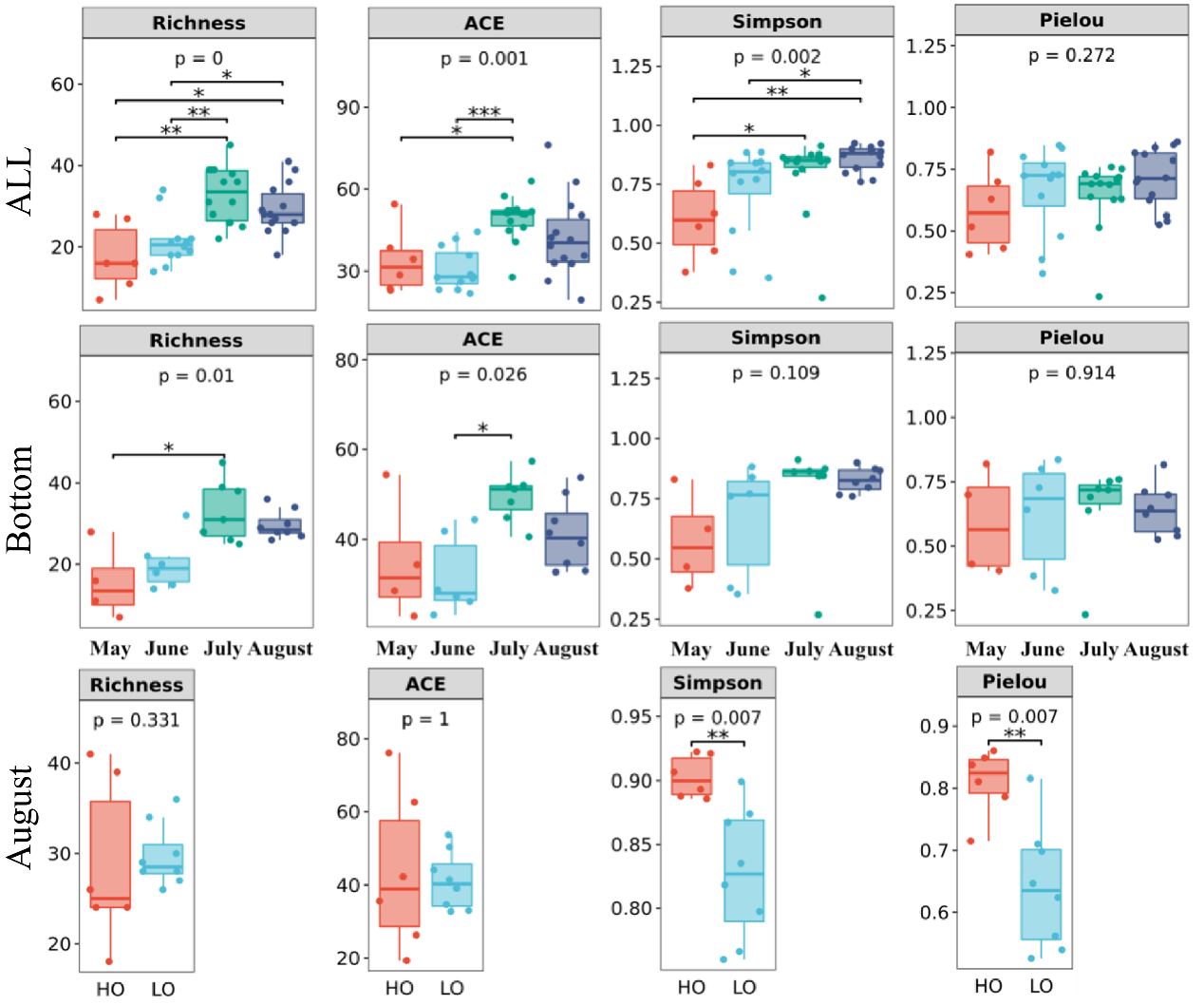


**Fig. S2** Box plots of alpha-diversity of the pathogenic bacterial communities in different groups in the Bohai Sea.


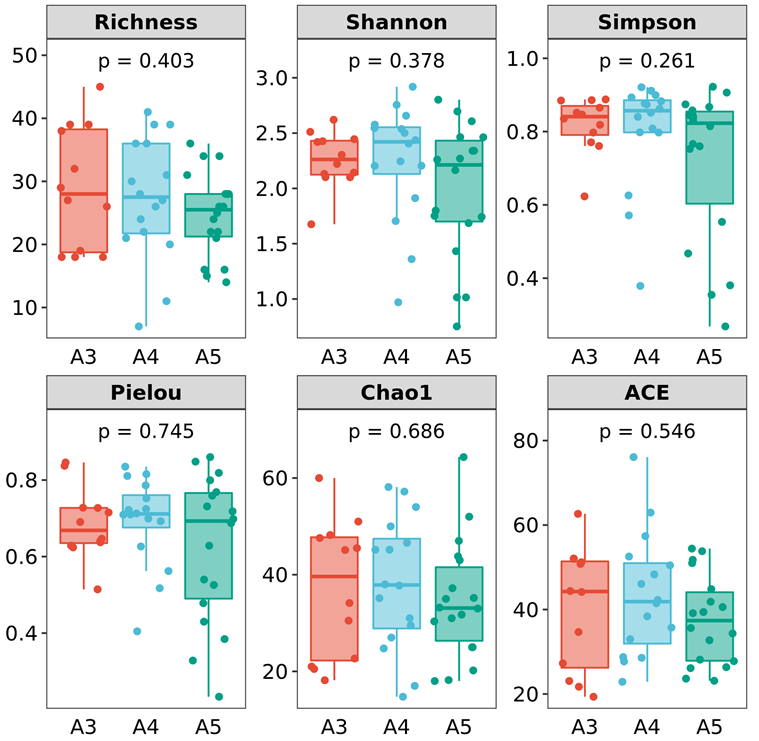


**Fig. S3** Box plots of alpha-diversity of the pathogenic bacterial communities in different site in the Bohai Sea.


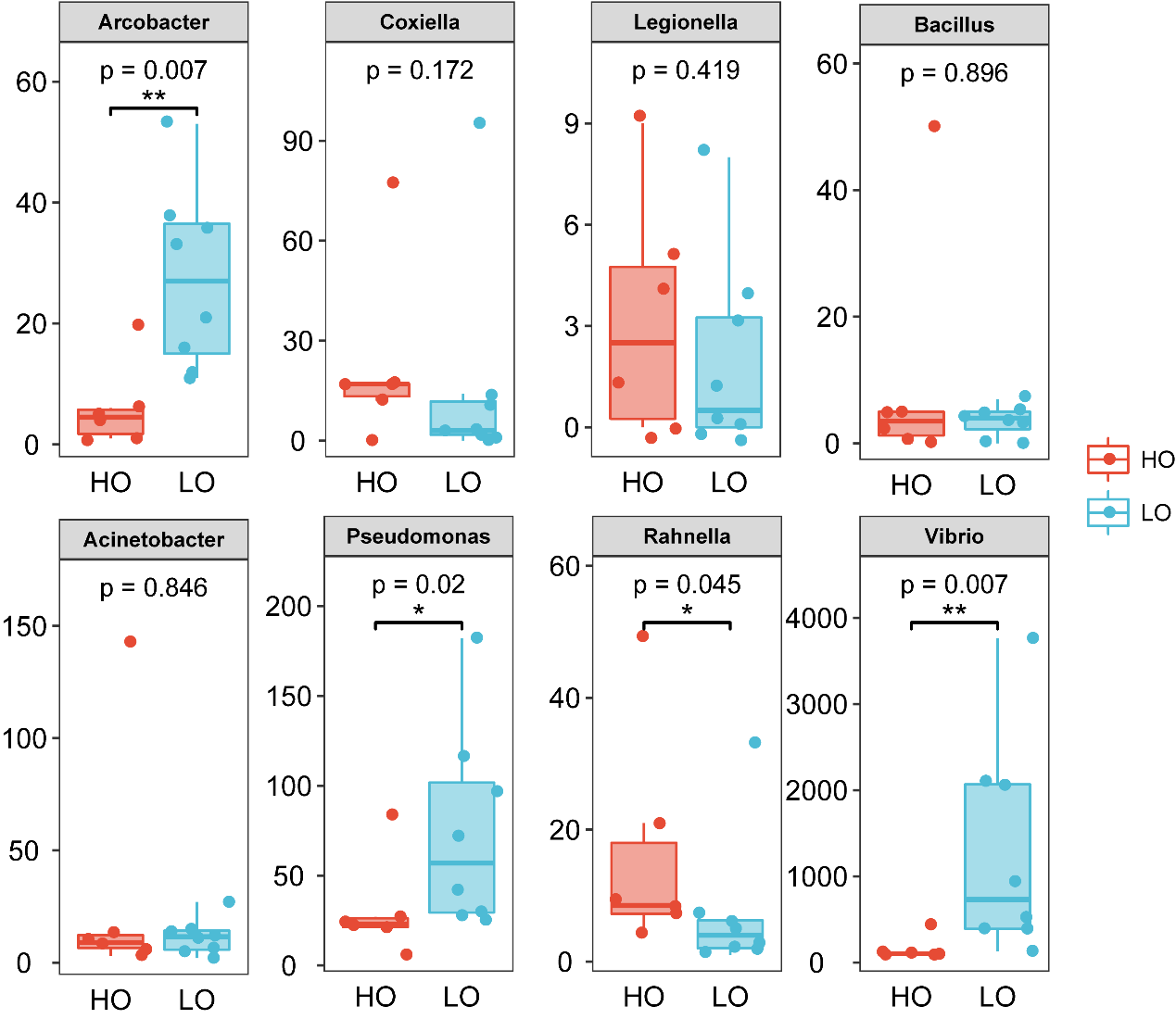


**Fig. S4**. Box plots of the abundance of pathogenic bacteria in samples with different oxygen concentrations in August in the Bohai Sea.

| **Samples** | **Variables** | **Mantel tests** | **Partial Mantel tests control factors** | |
| --- | --- | --- | --- | --- |
|  |  |  | **Depth** | **Distance** |
| June | Phosphate | **0.3282** | **0.3384** | **0.3319** |
|  | Nitrite | **0.3619** | **0.38** | **0.362** |
| July | Nitrate | **0.4084** | **0.4072** | 0.3039 |
|  | Nitrite | **0.4267** | **0.426** | 0.352 |
|  | Salinity | **0.4017** | **0.4** | 0.302 |
| August | DO | **0.3295** | 0.06477 | **0.3282** |
|  | Phosphate | **0.3496** | 0.0681 | **0.3485** |
|  | Nitrate | **0.2478** | 0.2121 | **0.2823** |
|  | Silicate | **0.3067** | -0.09735 | **0.3056** |

**Table S1** Mantel and partial Mantel tests for the Spearman's rank correlations between environmental parameters and beta-diversity. Tests were performed separately using all samples, and samples from different months, respectively. Bold correlations were corrected by Benjamini-Hochberg multiple tests when the p-value was less than 0.05.
